# Modified pectoralis major transfer: a single-incision technique for scapular winging

**DOI:** 10.1016/j.xrrt.2026.100845

**Published:** 2026-08-05

**Authors:** Miloš Spasojevic, Rory Cuthbert, Jeffery S. Hughes

**Affiliations:** Sydney Shoulder Research Institute, St Leonards, Australia

**Keywords:** Scapular winging, Serratus anterior palsy, Long thoracic nerve palsy, Pectoralis major transfer, Allograft, Suspensory fixation

Scapular winging due to serratus anterior dysfunction after long thoracic nerve palsy disrupts scapulothoracic mechanics and may lead to pain, fatigue, limited overhead function, and impaired activities of daily living.^6^ In patients with persistent symptoms despite structured rehabilitation, pectoralis major transfer remains a commonly used reconstructive option.^7^^,^^21^^,^^25^

Multiple technical variations of pectoralis major transfer have been described since the original report by Tubby.^12^^,^^27^

Accurate diagnosis can be challenging, and misdiagnosis may lead to unnecessary procedures and prolonged morbidity; therefore, electrodiagnostic confirmation of long thoracic nerve dysfunction and careful exclusion of alternative etiologies are essential before proceeding to tendon transfer.^29^

Contemporary techniques can be broadly conceptualized as direct transfers (tendon fixed directly to the scapula) and indirect transfers (tendon extended with an interpositional graft). Direct fixation can provide a short construct but may require substantial tension at fixation, which can risk post-operative stiffness, discomfort, and fixation compromise.^26^ This concern has been described in direct transfer series that emphasize the importance of avoiding overconstraint.^26^ Indirect techniques can facilitate positioning and tensioning but may be susceptible to graft elongation over time, contributing to recurrent winging. In addition, many established methods use dual-incision exposure (anterior harvest and posterior scapular fixation), which can increase approach-related morbidity.^3^^,^^18^

The purpose of this article is to describe a single-incision modified pectoralis major transfer that uses allograft augmentation integrated into the transferred tendon and knotless transosseous suspensory fixation at the inferior angle of the scapula.

The novelty of the present technique lies in the combination of anterior-only exposure, tibialis anterior allograft reinforcement incorporated within the transferred pectoralis major tendon, and adjustable knotless transosseous suspensory fixation at the inferior angle of the scapula. Unlike traditional indirect transfers, the allograft is used primarily as internal reinforcement rather than as a long interpositional lengthening graft, which may theoretically reduce elongation over time; however, this has not been biomechanically validated. To our knowledge, this exact combination has not previously been described.

The technique is designed to (1) avoid a posterior incision, (2) enable controlled, incremental intraoperative tensioning under direct visualization, and (3) minimize the length of exposed graft.

We present operative setup, key technical steps, and a standardized rehabilitation protocol.

## Anatomy

The pectoralis major is a broad, fan-shaped muscle composed of clavicular and sternal heads that converge to form a single tendon inserting onto the lateral lip of the intertubercular sulcus of the humerus. The sternal head contributes the majority of the tendinous insertion, whereas the clavicular head is predominantly muscular at its humeral attachment. With the arm in neutral position, the tendon undergoes a characteristic torsion, such that the inferior fibers of the sternal head insert superior and deep relative to the clavicular fibers.^10^^,^^23^

This torsional anatomy permits selective mobilization of the sternal head while preserving the clavicular head, allowing transfer without the need for repair or reinsertion of the clavicular component. Preservation of the clavicular head is important to maintain cosmesis and residual pectoralis major function.

The serratus anterior originates from the first through ninth ribs and inserts along the medial border of the scapula, acting as a primary stabilizer of the scapula against the thoracic wall. Dysfunction results in medial scapular winging and impaired scapulothoracic rhythm.^6^ The inferior angle and lateral border of the scapula provide a robust osseous target for fixation, with sufficient cortical thickness to allow transosseous tunneling.^6^

Access to the inferior angle of the scapula through an anterior axillary window places the chest wall and serratus anterior medially and the brachial plexus laterally. The subscapularis arises from the anterior surface of the scapula and may be bluntly split near the inferior angle to allow direct visualization of the lateral scapular border for controlled drilling and fixation.

### Anatomic pearl

Fixation should be placed through the thickened lateral border of the inferior scapular angle to optimize purchase and minimize risk of fracture.

## Indications

• Symptomatic medial scapular winging due to established long thoracic nerve palsy.
• Persistent functional limitation despite a minimum of 6 months of supervised, compliant physiotherapy.
• Clinical evidence of serratus anterior dysfunction, including a positive scapular assistance test.^14^
• Electrophysiologic confirmation of long thoracic nerve impairment.
• Imaging (magnetic resonance imaging [MRI]) excluding alternative or secondary causes of scapular winging and demonstrating serratus anterior atrophy or fatty infiltration when present.
• Passively reducible scapular winging.

Although a minimum of 6 months of supervised physiotherapy is used as an initial threshold for surgical consideration, treatment decisions are individualized according to symptom duration, functional limitation, etiology, clinical progression, and electrodiagnostic findings. Electrophysiologic assessment is used to confirm long thoracic nerve dysfunction and evaluate evidence of reinnervation or persistent denervation. Repeat electromyography may be useful when ongoing neurologic recovery remains possible. In patients with documented iatrogenic injury and persistent symptomatic winging without evidence of recovery, earlier surgical consideration may be appropriate.

## Contraindications

- Inability or unwillingness to comply with post-operative immobilization and rehabilitation.
- Fixed, passively irreducible scapular winging (advanced chronicity with stiffness or spasticity).
- Active infection.
- Relative:
- Prior axillary surgery or radiation with anticipated scarring that may compromise safe anterior access.
- Concomitant neuromuscular disorders affecting scapular stabilizers.

## Technique

• Patient positioning and setup (Video 1)
• Superficial exposure, sternal head identification, and release
• Graft preparation
• Subscapular access and scapular preparation
• Transosseous fixation and tensioning
• Closure
• Post-operative rehabilitation

### Patient positioning and setup

The procedure is performed under general anesthesia. A supplemental regional block may be administered at the discretion of the operating surgeon. Standard intravenous antibiotic prophylaxis is given prior to incision.

The patient is positioned in a 20° beach-chair position with the operative arm supported in a hydraulic arm holder. The entire shoulder girdle, axilla, and anterior chest wall are prepared and draped in a sterile fashion. Care is taken to allow lateral translation and retraction of the scapula, facilitating anterior access to both the pectoralis major tendon and the inferior angle of the scapula without the need for a posterior incision.

Fluoroscopy is positioned to allow intraoperative confirmation of suspensory device deployment at the inferior angle of the scapula.

#### Pearl

Adequate scapular mobility on the chest wall is essential to enable safe anterior access to the inferior scapular angle.

#### Pitfall

Inadequate positioning may necessitate an unplanned posterior incision.

### Superficial exposure, sternal head identification, and release

A standard deltopectoral approach is used. The deltopectoral interval is identified and developed, with protection of the cephalic vein. The clavipectoral fascia is incised, and the pectoralis major tendon is exposed at its humeral insertion.

**Figure 1:**
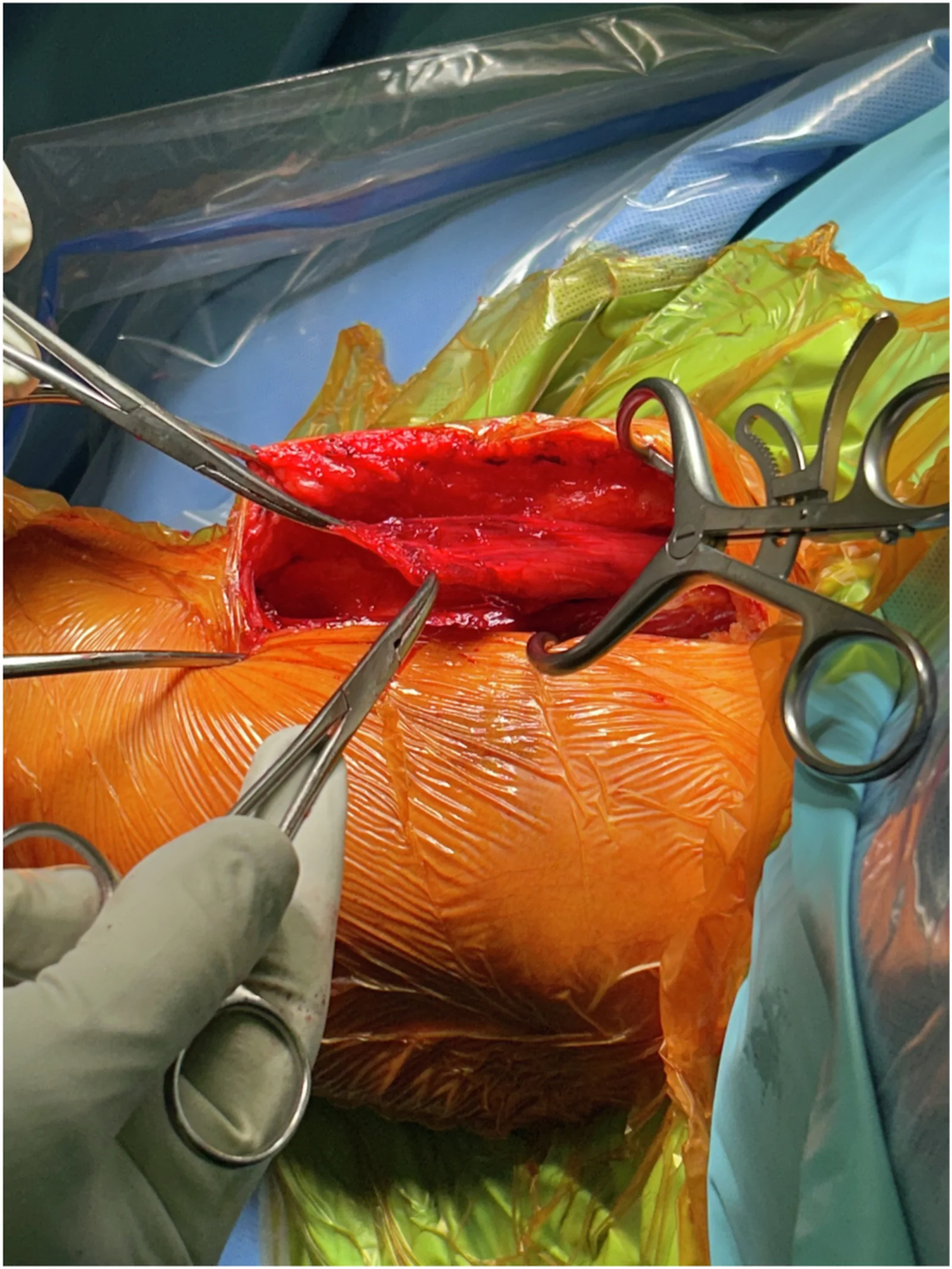
Deltopectoral exposure and identification of the pectoralis major tendon. The deltopectoral interval is developed with protection of the cephalic vein, exposing the humeral insertion of the pectoralis major tendon.

**Figure 2:**
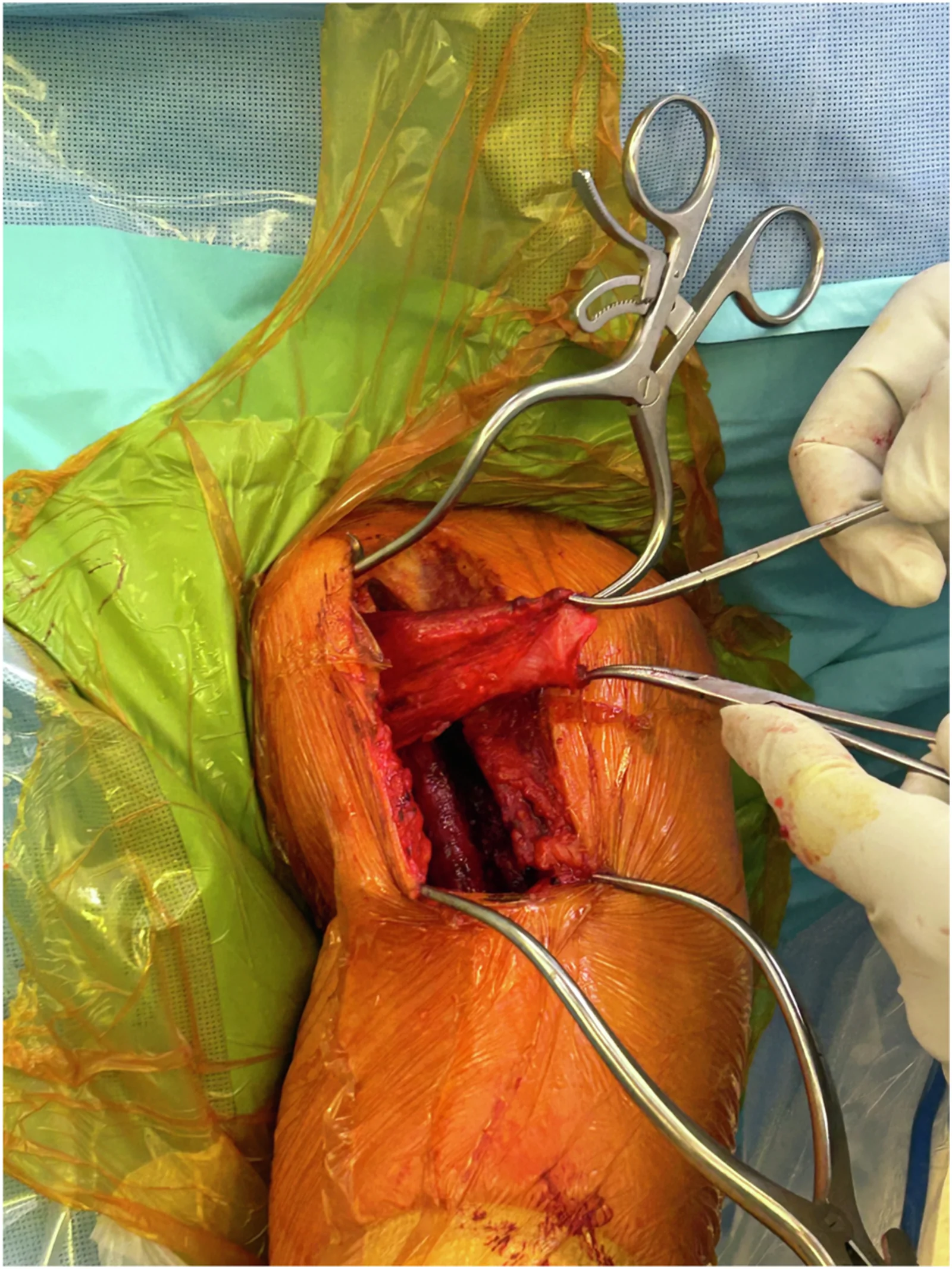
Identification of the sternal and clavicular heads of the pectoralis major. The intermuscular plane created by the native torsion of the tendon allows selective mobilization of the sternal head while preserving the clavicular head.

The superior and inferior border of the pectoralis major are identified. The bony landmarks of the sternal head of the pectoralis major are identified and these guide the dissection of the sternal head from the clavicular head. The pectoralis major tendon is exposed. With the arm in slight abduction and flexion, the intermuscular groove resulting from the torsion of the sternal head under the clavicular head can be visualized and dissected. The sternal head is dissected sub-periosteally at its far footprint and released (Figs. 1 and 2; pectoralis harvest).

Protection of the clavicular head is essential to avoid unnecessary repair/reinsertion, avoiding deltopectoral groove deepening and thus preserving pectoralis major function and cosmesis.

**Figure 3:**
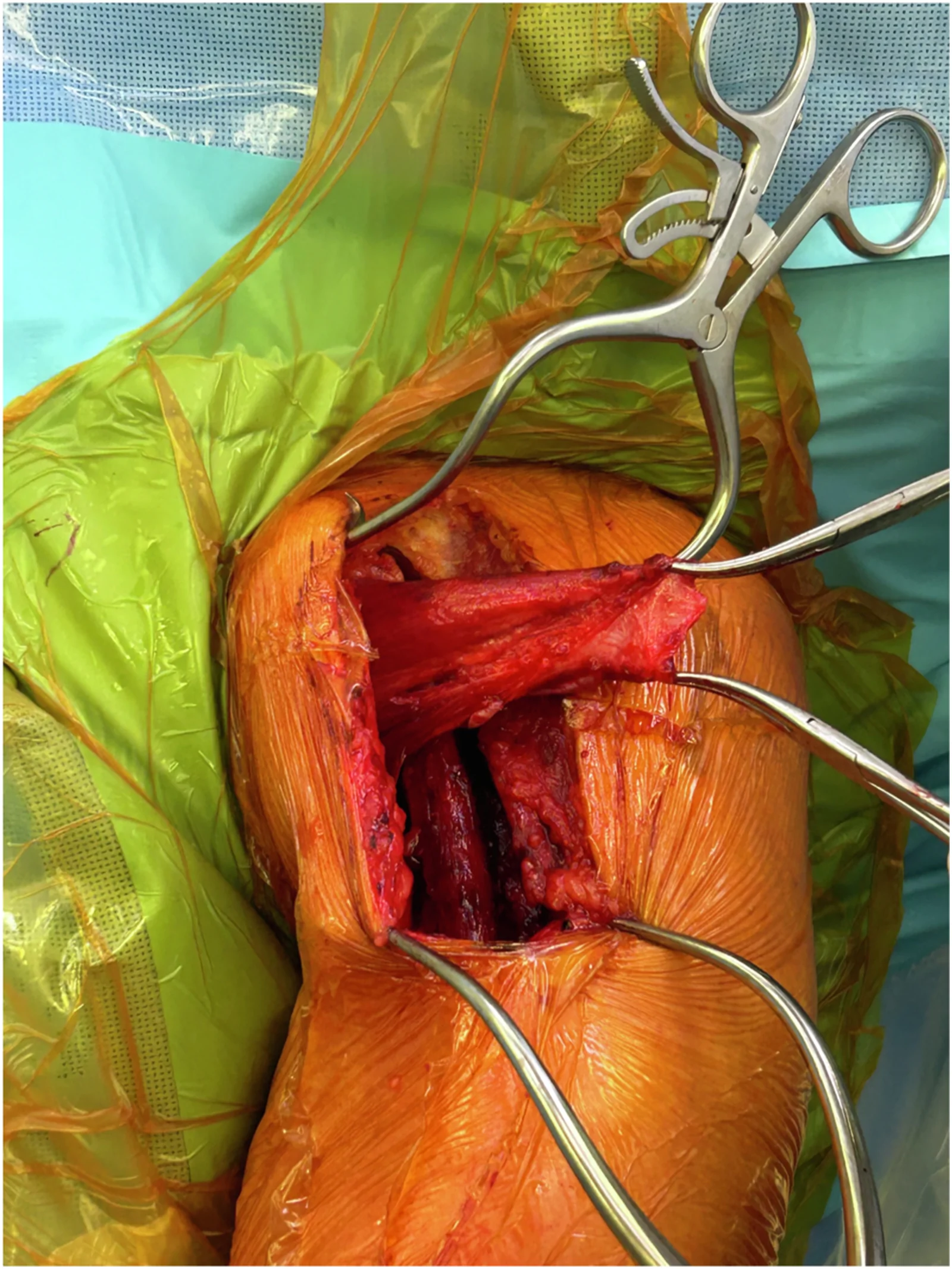
Assessment of sternal head excursion following release. After sub-periosteal release at the humeral footprint, the sternal head demonstrates adequate horizontal excursion without tension on the clavicular head.

Sternal head tendon excursion is visually confirmed, the neurovascular supply is posterior but does not need to be dissected. In our experience, complete release is suggested when horizontal excursion of the sternal head reaches approximately 5 cm without transmitting tension to the clavicular head (Video 2, pectoralis excursion, Figure 3, pectoralis excursion).

#### Pearl

Preservation of the clavicular head avoids deltopectoral groove deepening and maintains pectoralis major cosmesis and residual function.

#### Pitfall

Inadvertent injury to the clavicular head may require repair and increase post-operative morbidity.

### Graft preparation and tendon augmentation

A tibialis anterior allograft was selected because it provides a consistent diameter, substantial tensile strength, and sufficient length for tendon reconstruction while avoiding donor-site morbidity associated with autograft harvest.^19^ Furthermore, its tubular morphology facilitates incorporation within the transferred pectoralis major tendon and straightforward integration with the suspensory fixation construct.

The graft is doubled over a knotless suspensory fixation device (ToggleLoc, Zimmer Biomet, Warsaw, IN, USA), ensuring free passage of the adjustable loop through the graft.

**Figure 4:**
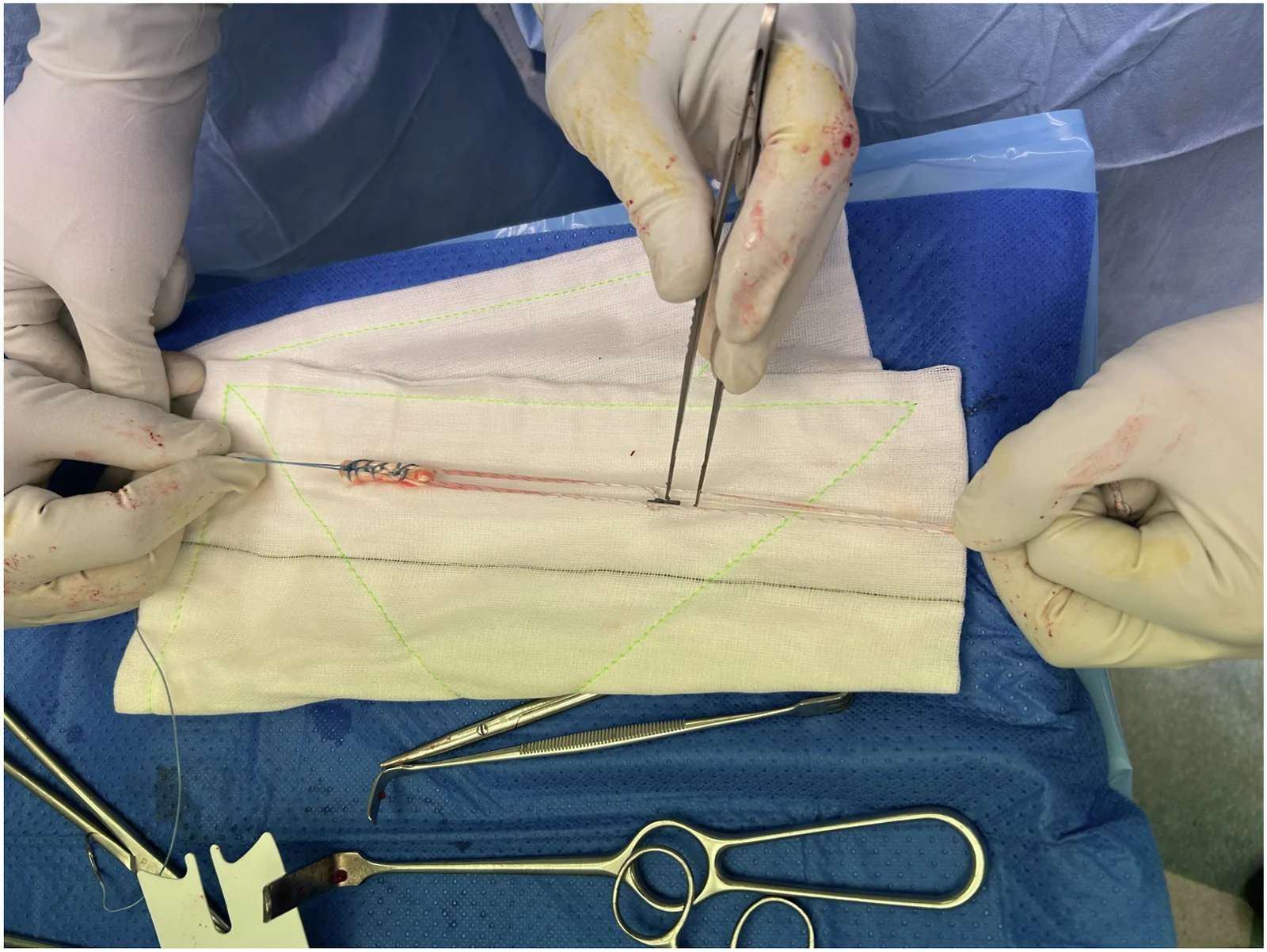
Preparation of the tibialis anterior allograft with suspensory fixation device. The allograft is doubled over a knotless adjustable-loop suspensory device, ensuring free sliding of the loop prior to incorporation.

**Figure 5:**
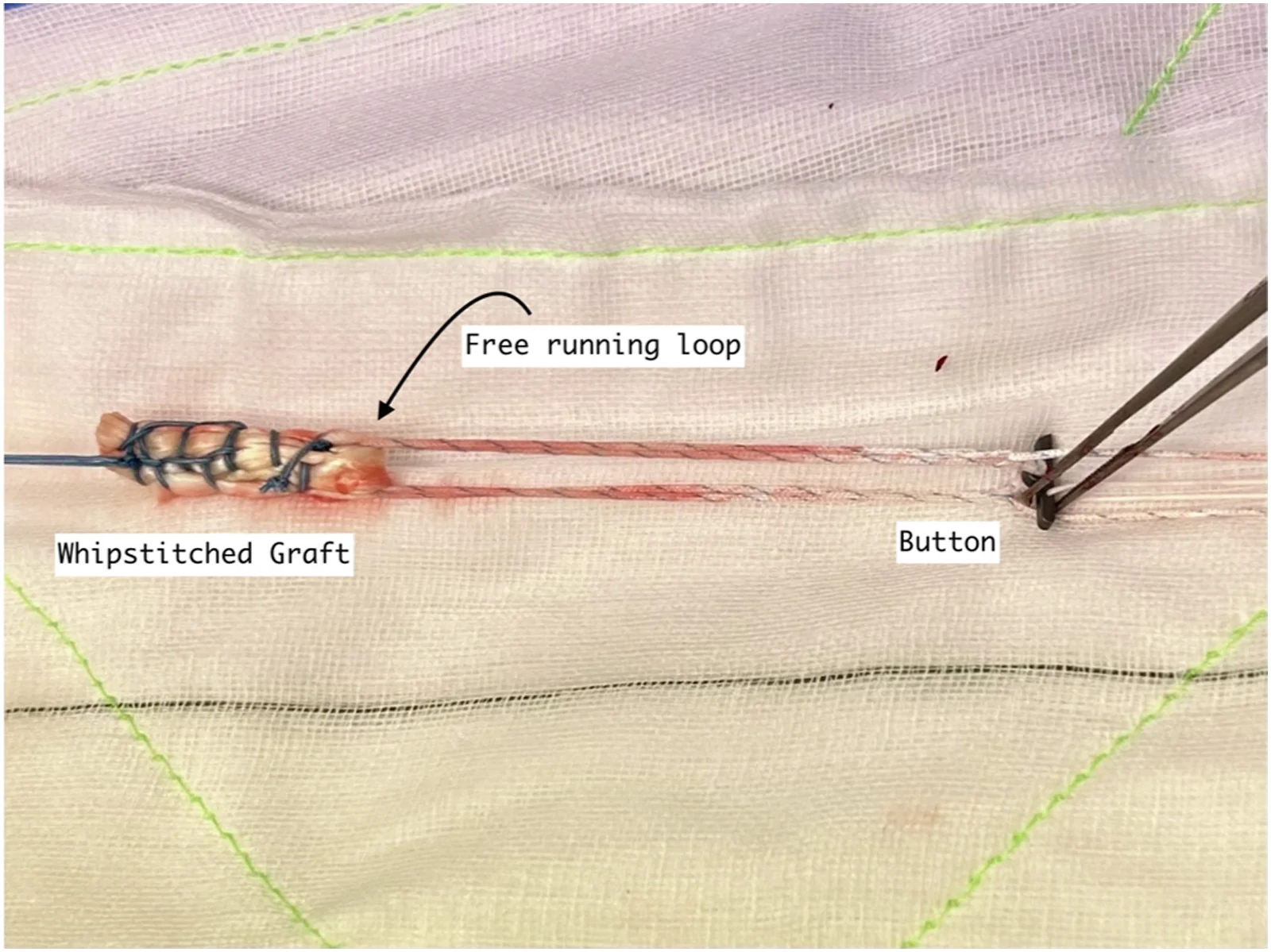
Completed allograft–suspensory construct. The doubled graft is whipstitched using non-absorbable sutures to create a compact reinforcement construct of uniform diameter.

The doubled graft is whipstitched using a Krakow-type whipstitch with a non-absorbable high-strength suture, creating a construct approximately 7 cm in length and 7 mm in diameter. Free sliding of the suspensory loop is confirmed prior to incorporation (Figs. 4 and 5; graft preparation).

The released sternal head of the pectoralis major tendon is then tubularized around the allograft, integrating the graft within the tendon substance. At least 5 cm of the allograft is fully covered by the native tendon using non-absorbable sutures, minimizing the length of exposed graft.

**Figure 6:**
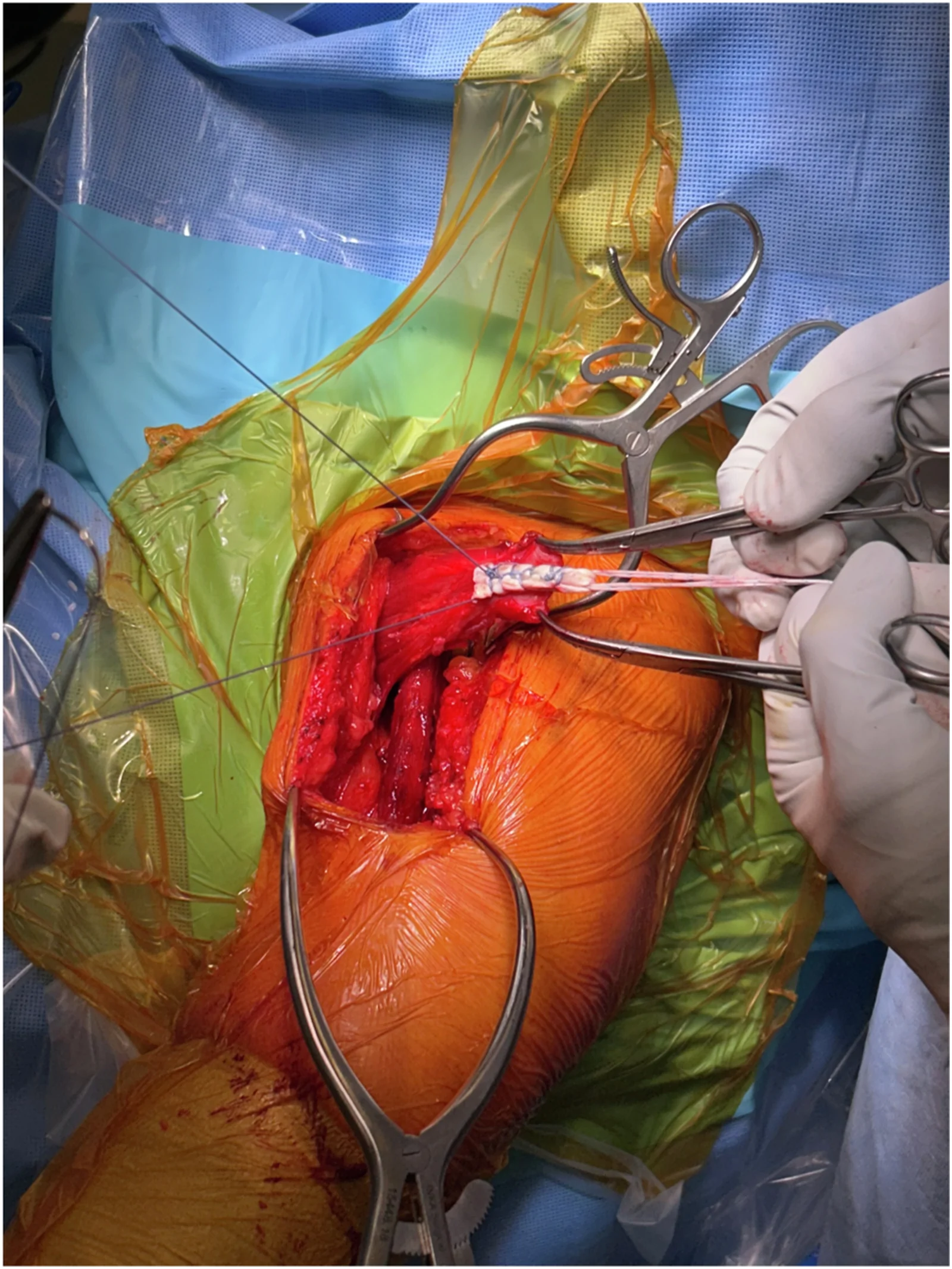
Integration of the allograft within the sternal head of the pectoralis major. The released sternal head is tubularized around the allograft, incorporating the graft within the native tendon substance.

**Figure 7:**
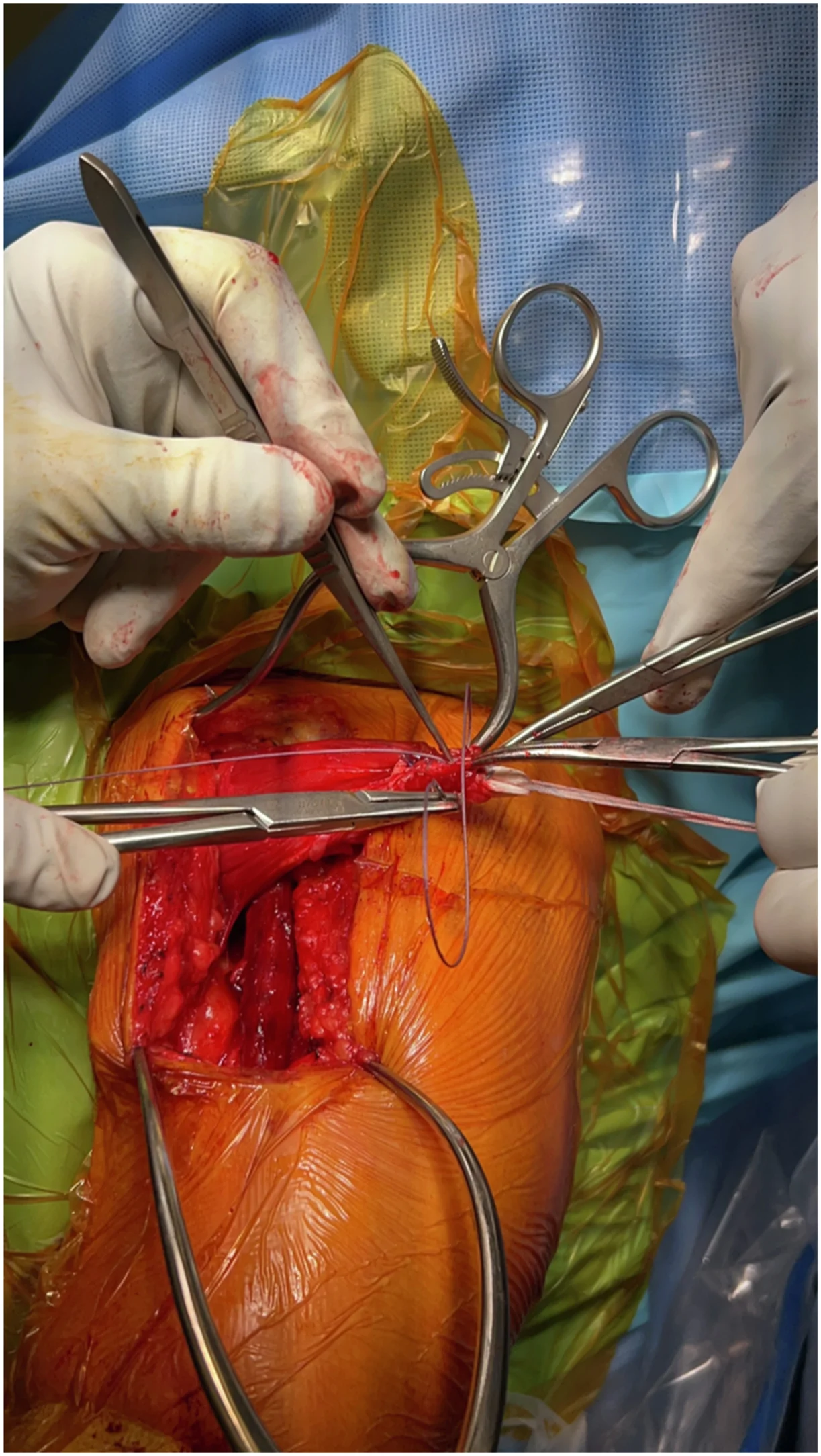
Tubularized tendon–allograft construct. At least 5 cm of the allograft is fully covered by the native tendon to minimize exposed graft length.

**Figure 8:**
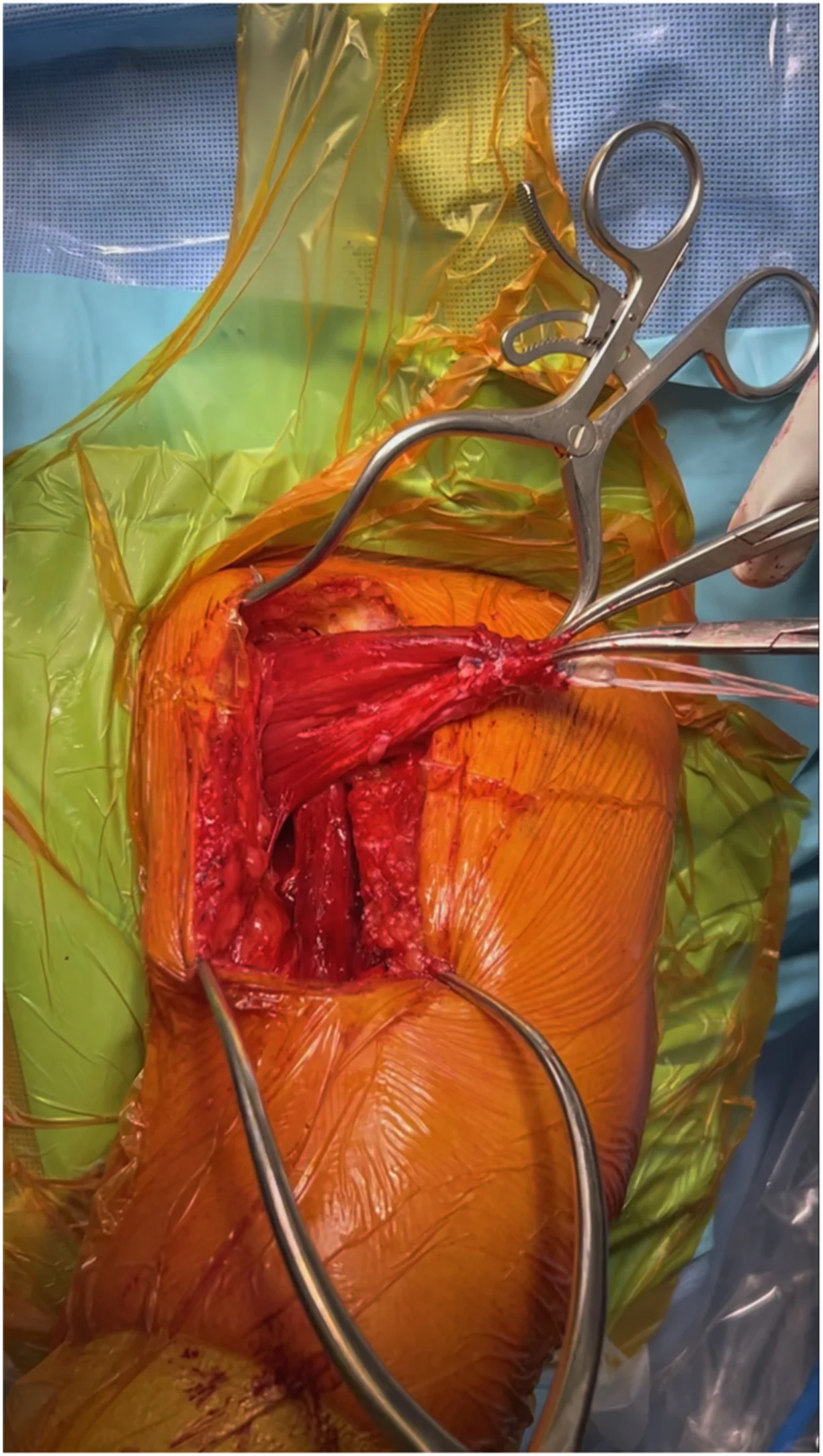
Final reinforced pectoralis major transfer construct. The completed construct functions as a reinforced tendon rather than a lengthened interpositional graft.

This augmentation is intended to reinforce the transferred tendon and limit the amount of free graft susceptible to elongation, rather than to increase the overall length of the transfer (Figure 6, Figure 7, Figure 8, graft tubularization).

#### Pearl

The allograft functions as an internal reinforcement; it should not significantly lengthen the construct.

#### Pitfall

Excessive exposed graft length may predispose the construct to elongation over time.

### Subscapular access and scapular preparation

Following graft augmentation, deep exposure to the inferior angle of the scapula is obtained through an anterior sub-pectoral window. The clavipectoral fascia is opened inferior to the pectoralis major, and blunt dissection is carried medially along the chest wall.

**Figure 9:**
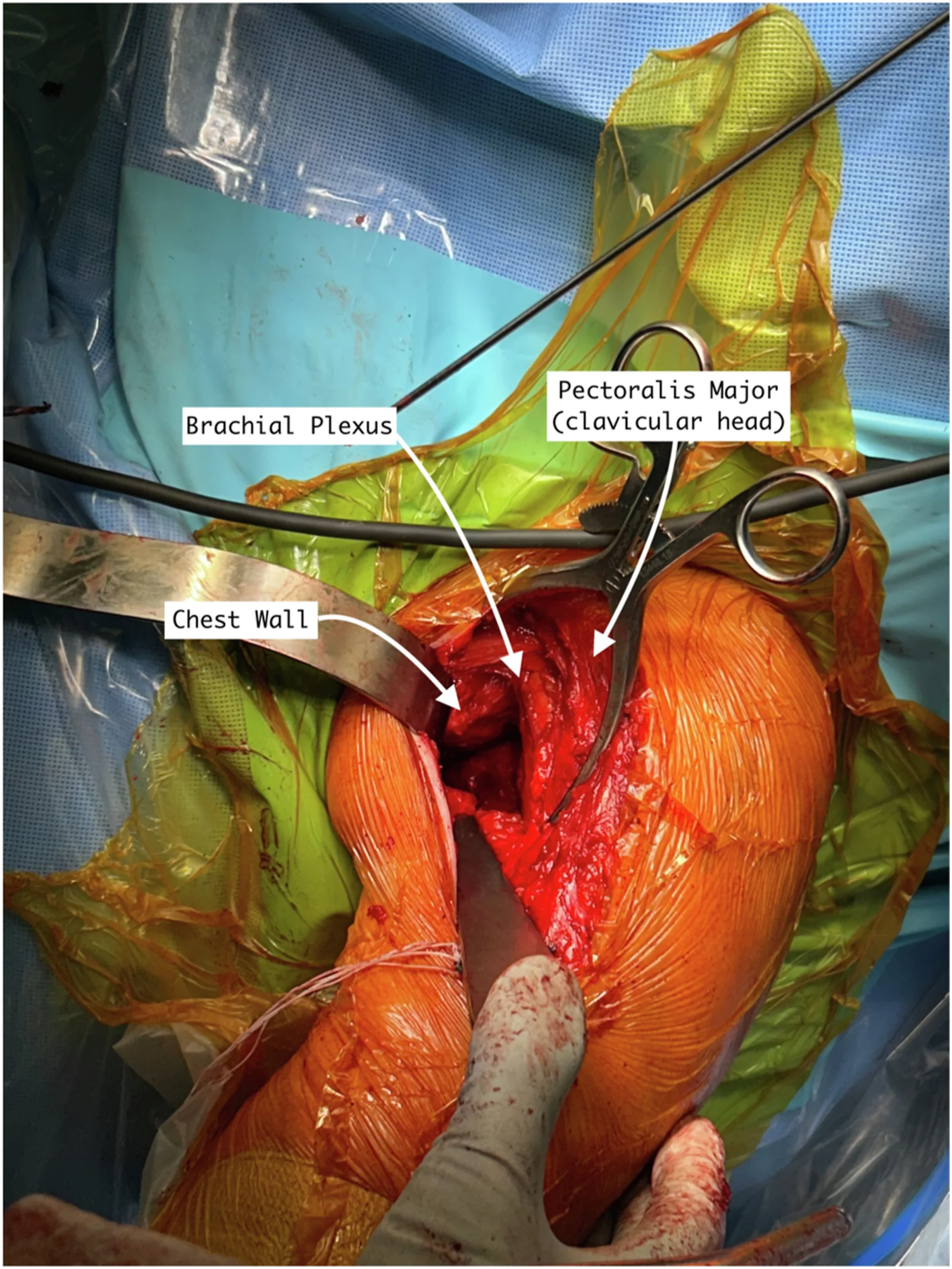
Anterior sub-pectoral access to the inferior angle of the scapula. Blunt dissection through an anterior window allows visualization of the serratus anterior and exposure of the thickened lateral border of the inferior scapular angle while protecting the brachial plexus.

The brachial plexus and axillary neurovascular structures are protected laterally, while the chest wall and serratus anterior are identified medially. A key principle of the anterior access is to avoid deep sharp dissection toward the inferior angle of the scapula. Instead, after the anterior sub-pectoral window is developed, the dissection is performed bluntly along the chest wall. A bone hook is placed around the inferior border of the scapula, allowing the scapula to be controlled and delivered toward the operative field. This maneuver reframes the exposure: rather than dissecting deeply toward the scapula, the scapula is mobilized toward the surgeon. Broad, blunt abdominal retractors are then used to maintain the working window and protect the chest wall, serratus anterior, brachial plexus, and axillary neurovascular structures. Fixation is directed to the thickened lateral border of the inferior scapular angle, where bone stock is greatest. (Fig. 9, access). The inferior subscapularis is split bluntly at its most distal aspect only as much as required to expose this lateral border under direct visualization. The inferior angle and lateral border are cleared of soft tissue using a periosteal elevator, allowing direct visualization of the bony surface.

#### Pearl

Direct visualization of the lateral scapular border is essential to ensure safe drilling and accurate implant placement. Mobilize the scapula toward the operative field rather than dissecting deeply toward the scapula.

#### Pitfall

Sharp medial dissection or narrow retractors may increase risk to the chest wall, serratus anterior, and neurovascular structures.

### Transosseous fixation and tensioning

Drilling is performed only after direct visualization of the thickened lateral border of the inferior scapular angle. The scapula is stabilized with a bone hook or narrow clamp to provide counterpressure. A standard 2 mm guidewire is placed through the lateral border at the planned fixation site, and a 4.5 mm cannulated drill is advanced in a controlled bicortical fashion. Care is taken to maintain perpendicular orientation relative to the scapular surface to optimize cortical support. Eccentric drilling, inadequate cortical bridge, or malposition may predispose to scapular fracture, inadequate cortical purchase, button maldeployment, or thoracic injury.

**Figure 10:**
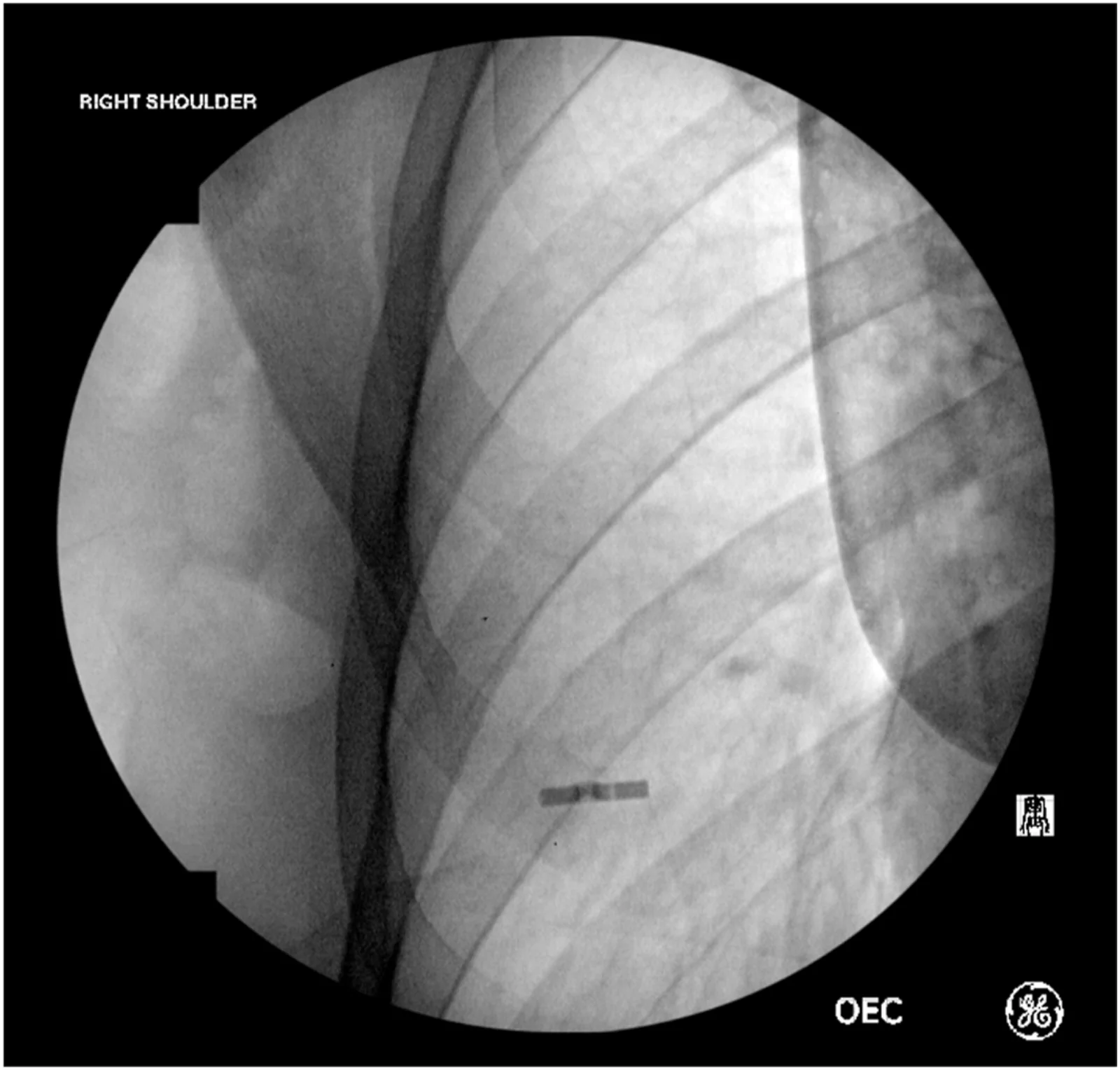
Intraoperative anteroposterior fluoroscopic confirmation of suspensory fixation. Fluoroscopy confirms appropriate deployment and seating of the cortical button on the inferior pole and lateral cortex of the scapula following transosseous passage.

**Figure 11:**
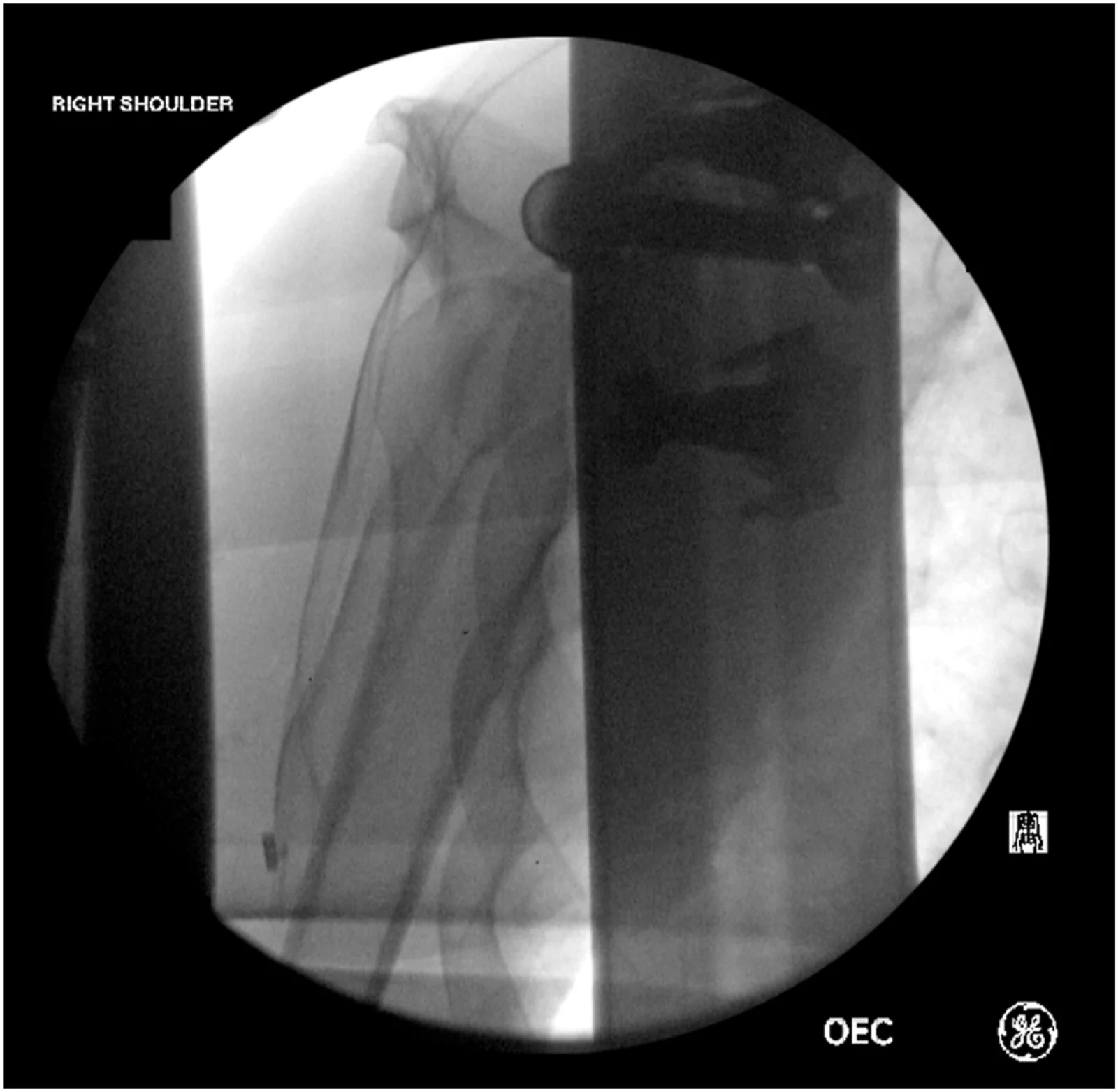
Intraoperative lateral fluoroscopic confirmation of suspensory fixation. Fluoroscopy confirms appropriate deployment and seating of the cortical button on the posterior cortex of the scapula following transosseous passage.

The knotless suspensory fixation device is then passed through the transosseous tunnel. The cortical button is deployed and flipped on the posterior cortex of the scapula. Correct deployment is confirmed visually and with intraoperative fluoroscopy, which is routinely used by the authors and strongly recommended (Figs. 10 and 11 intraoperative fluoroscopy).

#### Pearl

Fluoroscopic confirmation of button deployment reduces the risk of incomplete cortical seating.

#### Pitfall

Inadequate cortical purchase or eccentric drilling may predispose to scapular fracture or fixation failure.

### Graft reduction and tensioning

Once the suspensory device is secured, the augmented pectoralis major tendon is reduced toward the inferior angle of the scapula. Using the adjustable loop mechanism, incremental tensioning is performed under direct visualization.

**Figure 12:**
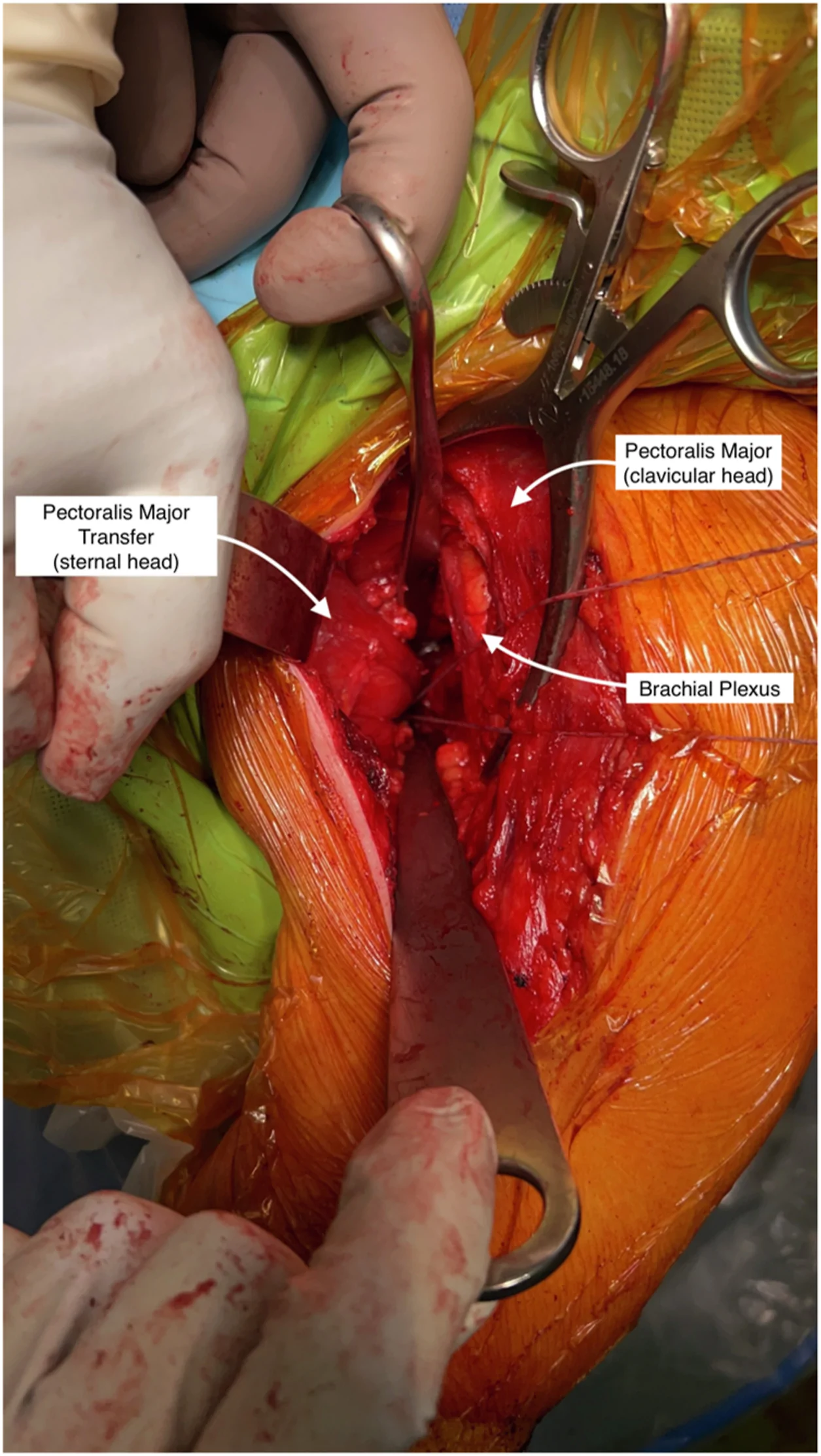
Final reduction and tensioning of the pectoralis major transfer. Incremental tensioning of the adjustable loop reduces medial scapular winging while preserving passive scapulothoracic mobility.

Tension is adjusted to achieve reduction of medial scapular winging while maintaining passive scapulothoracic mobility. The construct allows controlled fine-tuning without the need for knot tying or posterior dissection (Video 3, tensioning, Fig. 12 transfer).

Final tensioning is performed with the arm in neutral rotation and slight forward flexion to avoid overconstraint.

#### Pearl

Progressive tensioning under direct visualization minimizes the risk of overtensioning.

#### Pitfall

Excessive tension may restrict scapulothoracic motion and contribute to post-operative stiffness or discomfort.

## Closure

After final tensioning is confirmed, the surgical field is irrigated thoroughly. Hemostasis is ensured, with particular attention to the sub-pectoral and axillary spaces. The surgical site is closed by routine absorbable subcutaneous and skin sutures.

A drain is not routinely required but may be considered at the surgeon's discretion in cases of extensive dissection or concern for post-operative hematoma.

The arm is placed in a shoulder immobilizer prior to emergence from anesthesia.

### Post-operative rehabilitation

Post-operative rehabilitation is standardized and coordinated with an experienced physiotherapist familiar with scapulothoracic mechanics and tendon transfer principles.

The arm is immobilized in a sling with an abduction pillow for six weeks, with removal permitted for hygiene and supervised exercises. During this period, hand, wrist, and elbow range-of-motion exercises are commenced immediately. Gentle pendulum exercises and supervised passive shoulder motion are permitted in the supine position with scapular stabilization, limited to 90° of forward elevation and 20° of external rotation. Active shoulder elevation, resisted internal rotation or adduction, pushing, weight bearing through the arm, and active scapular strengthening are avoided.

At six weeks, the sling is discontinued and active-assisted followed by active shoulder range of motion is initiated and progressively advanced. The focus between 6 and twelve weeks is restoration of functional range of motion and scapular control, with light isometric activation introduced as tolerated.

From twelve weeks, progressive strengthening and neuromuscular re-education are commenced, emphasizing retraining of the transferred pectoralis major as a dynamic scapular stabilizer. Heavy lifting and unrestricted strengthening are avoided until six months post-operatively, with return to full activity typically permitted between 6 and nine months depending on functional recovery.^4^^,^^8^

#### Pearl

Emphasis on scapular control and gradual loading is critical to allow biological incorporation and adaptation of the transfer.

#### Pitfall

Premature strengthening may overload the construct and compromise fixation.

### Clinical experience

To date, we have performed 4 transfers in 4 patients (mean age 37 years, range 16–66 years) with a minimum follow-up of 24 months. There were no intraoperative neurovascular injuries, pleural injuries, scapular fractures, or fixation failures. One patient developed wound dehiscence with a superficial wound infection that resolved with oral antibiotic therapy without further surgical intervention.

Three patients underwent transfer for iatrogenic long thoracic nerve injury following previous surgery, whereas 1 patient had early fascioscapulohumeral muscular dystrophy predominantly affecting the serratus anterior. At latest follow-up, all patients demonstrated improvement in pain and clinical resolution of scapular winging. Patients with iatrogenic nerve injury showed sustained improvements in shoulder function and forward flexion. The patient with fascioscapulohumeral muscular dystrophy experienced progression of the underlying disease in other muscle groups during follow-up, although the transferred construct remained clinically functional.

These preliminary observations suggest that the described technique is feasible and may provide durable correction of medial scapular winging. However, larger prospective studies with validated outcome measures are required before conclusions regarding efficacy can be drawn.

Based on tendon transfer principles and experience, by the 8-week mark, patients typically demonstrate static effect of the tendon, whereas the dynamic effect will require longer rehabilitation (3-6 months).

**Figure 13:**
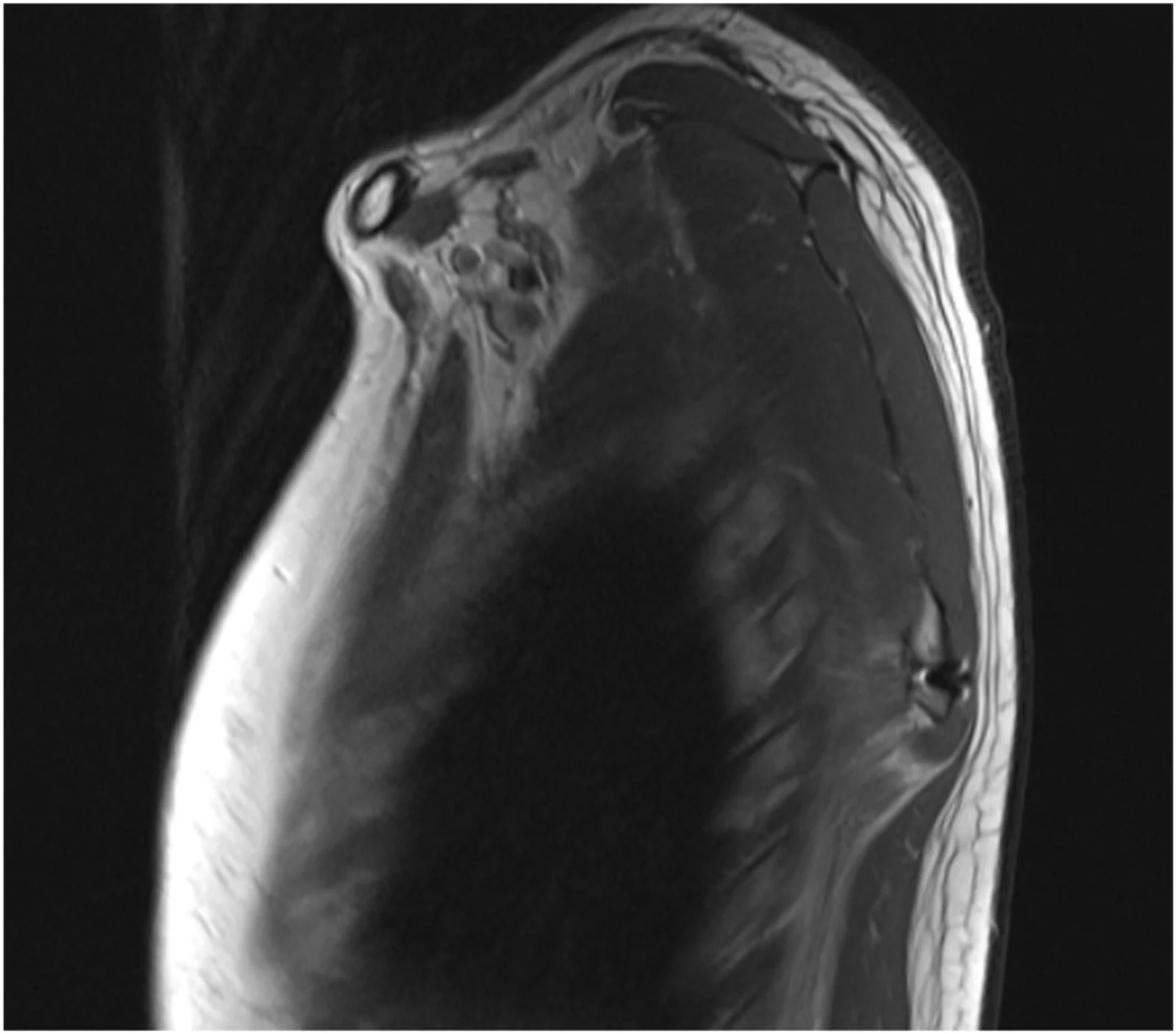
Sagittal T1-weighted magnetic resonance image of the thorax demonstrating the cortical button positioned at the inferior angle of the scapula following suspensory fixation.

**Figure 14:**
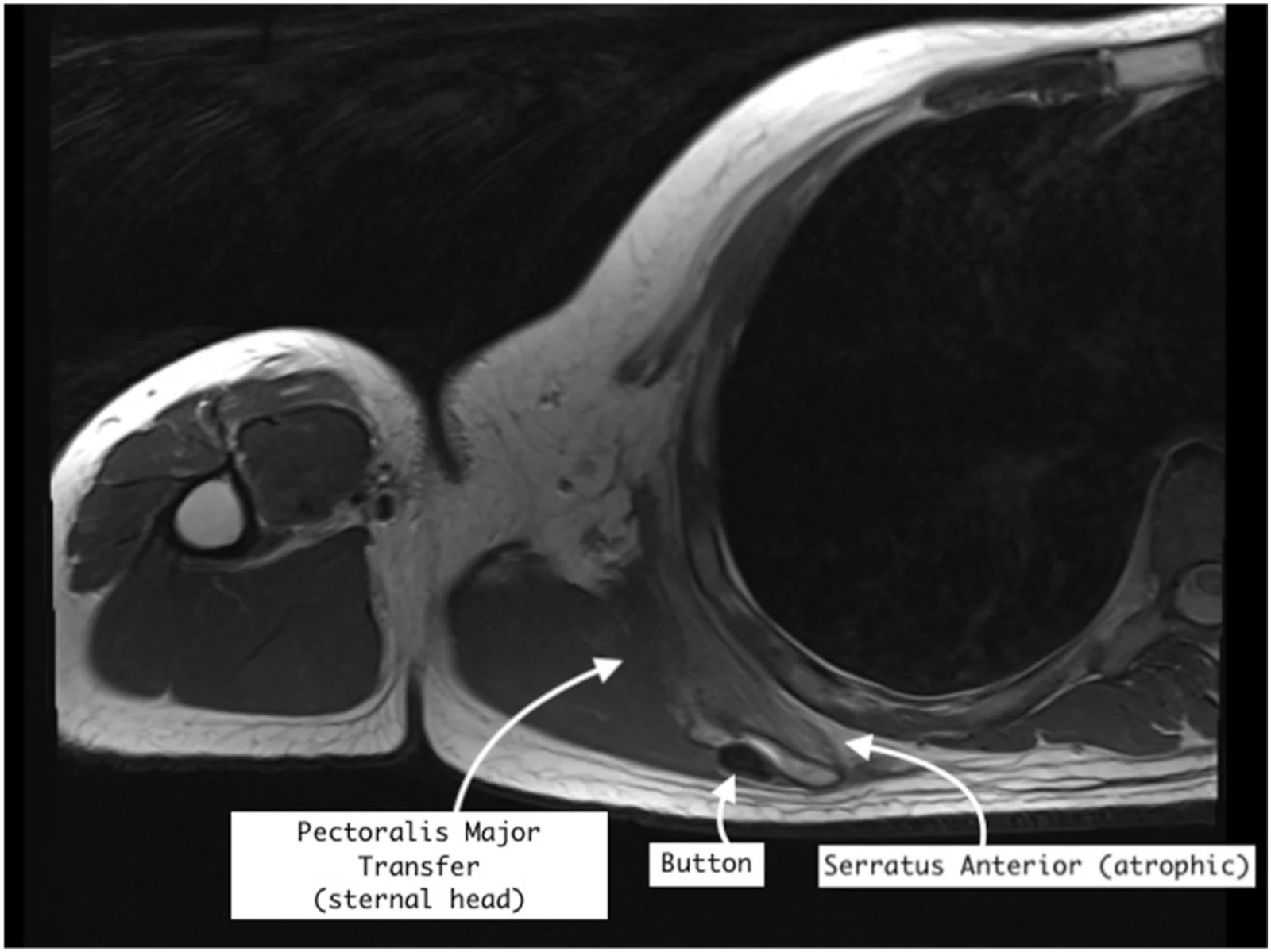
Axial T1-weighted magnetic resonance image of the thorax demonstrating the transferred pectoralis major tendon coursing toward its fixation at the inferior scapular angle.

Integration of the transfer can be confirmed with MRI imaging (Figs. 13 and 14 Post-operative MRI).

### Complications

- Failure of fixation
  ○ Persistent winging (asymptomatic)
  ○ Persistent winging (symptomatic with unresolved pain)
  ○ Tunnel malposition
  ○ Inadequate cortical purchase
  ○ Scapular fracture
  ○ Graft failure or elongation
- Neurovascular injury (brachial plexus, axillary vessels, or thoracodorsal structures)
- Thoracic or pleural injury
- Post-operative neurapraxia
- Overtensioning resulting in pain, stiffness, or restricted scapulothoracic motion
- Undertensioning resulting in persistent or recurrent winging
- Persistent shoulder dysfunction
- Cosmetic concerns
- Surgical site complications, including wound dehiscence, infection, seroma, or hematoma

## Discussion

The purpose of this article is to describe a single-incision modification of pectoralis major transfer for the treatment of medial scapular winging secondary to long thoracic nerve palsy. The technique is designed to address several commonly reported limitations of traditional pectoralis major transfer procedures, including approach-related morbidity, challenges with intraoperative tensioning, and recurrent winging associated with graft elongation or fixation failure.^3^^,^^5^^,^^20^^,^^25^

### Approach-related morbidity

Many established pectoralis major transfer techniques require combined anterior and posterior exposures to access the inferior angle of the scapula. While effective, posterior dissection has been associated with increased morbidity, including seroma formation, scar-related discomfort, risk to surrounding neurovascular structures, and post-operative stiffness.^9^^,^^17^^,^^24^ The single-incision anterior approach described in this report avoids a posterior incision by permitting access to the inferior scapular angle through a controlled sub-pectoral window. This approach preserves posterior scapular stabilizers and theoretically minimizes soft-tissue disruption, while still allowing direct visualization of the fixation site.

Although reported complication rates following pectoralis major transfer vary widely in the literature, many adverse events appear to be approach-related rather than construct-related.^5^^,^^9^^,^^26^ By limiting dissection planes and avoiding an additional surgical field, this technique is intended to reduce exposure-related morbidity while maintaining reliable access to the scapular fixation site.

Anatomic studies have demonstrated variability in the attachment of the latissimus dorsi and teres major at the inferior angle of the scapula, emphasizing their synergistic contribution to scapular stabilization.^1^^,^^22^ Avoiding posterior dissection preserves these structures, which may provide compensatory stabilization in cases of serratus anterior dysfunction.^8^^,^^15^^,^^28^ This consideration is particularly relevant given the dynamic nature of scapulothoracic motion and the goal of preserving residual stabilizing mechanisms whenever possible.

A comparative summary of adverse outcomes across key clinical series is provided in Table I, highlighting that complications cluster around (1) exposure-related morbidity, (2) graft-related morbidity or elongation in indirect constructs, and (3) neurologic symptoms or stiffness across approaches (Table I: Reported complications and adverse outcomes in published clinical series of pectoralis major transfer for scapular winging due to long thoracic nerve palsy).

**Table I:** Summary of reported complications and adverse outcomes following pectoralis major transfer for scapular winging.

Author (yr)	Technique	No. of shoulders	Graft (if indirect)	Fixation method	Overall complications (%)	Recurrent/residual winging (n)	Soft-tissue complications (n)	Neurologic complications (n)	Graft-related failure or elongation (n)	Stiffness/capsulitis/weakness (n)
Iceton^12^ (1987)	Indirect	15	Fascia lata	Not specified	57	–	3 (insertion scarring)	2 (pre-existing)	4 (1 avulsion, 3 scarring)	–
Post^21^ (1995)	Indirect	8	Fascia lata	Transosseous	25	2	2 (1 hematoma, 1 seroma)	–	–	–
Warner^29^ (1998)	Indirect	8	Semitendinosus and gracilis	Transosseous	25	1	1	–	–	–
Connor^5^ (1997)	Indirect	11	Fascia lata	Transosseous	9	1	–	–	–	–
Perlmutter^20^ (1999)	Indirect	16	Fascia lata	Transosseous	13	–	–	–	2 (elongation)	–
Steinmann^24^ (2003)	Indirect	9	Fascia lata	Transosseous	33	–	1 (thigh seroma)	–	–	2
Tauber^26^ (2008)	Direct	12	–	Direct apposition	17	–	–	1 (arm paresthesia)	1 (traumatic)	–
Galano^9^ (2008)	Direct	11	–	Onlay	18	–	1	–	1 (traumatic)	–
Streit^25^ (2012)	Mixed	26	Semitendinosus and gracilis	Onlay/transosseous	19	–	1 (hematoma)	4 (donor neurapraxia/paresthesia)	–	–
Noerdlinger^18^ (2002)	Indirect	15	Fascia lata	Not specified	27	–	–	1 (donor site)	–	3
Elhassan^7^ (2015)	Direct	51	–	Transosseous (bony)	24	–	7 (2 infections, 5 hematomas)	3 (sensory loss)	–	6
Chalmers^3^ (2014)	Mixed	14	Tibialis anterior/achilles	Transosseous	∼21	–	–	1 (CRPS)	2 (indirect)	1
Li^17^ (2017)	Indirect	28	Semitendinosus	Transosseous	18	–	1 (seroma)	–	–	4
Geurkink^11^ (2024)	Indirect	16	Achilles	Transosseous	31	5	–	–	–	–

*CRPS*, complex regional pain syndrome.

Data reflect heterogeneous surgical techniques, graft choices, fixation methods, and reporting standards across studies. Complication rates and failure definitions are reported as described by the original authors. Mixed series include both direct and indirect transfer techniques where specified.

Direct transfer indicates fixation of the pectoralis major tendon directly to the scapula without interposition graft.

Indirect transfer indicates the use of an interposition graft to extend the tendon.

Reported complication rates and adverse events are reproduced as described in the original studies; categorization was standardized for comparison.

Residual winging includes asymptomatic scapular prominence reported at the final follow-up.

These reported patterns informed the present modification, which aims to avoid posterior exposure, eliminate autograft harvest, minimize ‘free’ graft length, and allow incremental tensioning under visualization. Across both direct and indirect techniques, reported complications include donor-site morbidity, neuropathic symptoms, residual or recurrent winging, and soft-tissue complications, with considerable variability between series. Importantly, many reported adverse events appear related to surgical exposure, graft harvest, or graft elongation rather than fixation failure alone.^11^

### Graft augmentation and construct design

Recurrent scapular winging after indirect pectoralis major transfer has frequently been attributed to elongation of interpositional grafts.^17^^,^^20^ Elongation and donor-site morbidity associated with fascia lata–augmented constructs have been recognized for decades, motivating efforts to reduce exposed graft length and minimize harvest-related morbidity.^21^

In contrast to techniques that rely on long, exposed graft segments, the current modification integrates a tibialis anterior allograft within the tubularized sternal head of the pectoralis major tendon. This design minimizes the length of uncovered graft, which may theoretically reduce elongation over time.

The allograft is intended to function as an internal reinforcement rather than as a primary lengthening element. By incorporating the graft into the transferred tendon, the construct maintains a short working length while allowing controlled positioning at the scapular attachment site. Similar reinforcement principles have been applied in other reconstructive tendon procedures to enhance construct robustness without increasing excursion length.^2^

### Fixation and tension control

Achieving appropriate tension is critical to the success of tendon transfer procedures.^16^ Excessive tension may restrict scapulothoracic motion and contribute to post-operative pain or stiffness, whereas insufficient tension may result in persistent or recurrent winging. The use of a knotless suspensory fixation device allows incremental tensioning under direct visualization, facilitating fine adjustment without knot tying or posterior exposure.^4^

Although biomechanical data specific to this application are limited, suspensory fixation devices have demonstrated reliable fixation strength in other reconstructive settings, and the construct described here is intended to operate within physiological loading demands encountered during shoulder motion.^13^^,^^19^ This method enables the surgeon to balance scapular reduction with preservation of passive scapulothoracic mobility.

### Biological considerations

By integrating the allograft within the native pectoralis major tendon, the construct may benefit from proximity to vascularized tissue, which could theoretically support graft incorporation and durability. Unlike techniques that rely on long exposed interposition grafts, the allograft is largely enclosed within vascularized native tendon. Although direct evidence for this construct is unavailable, tendon healing studies have demonstrated that cellular repopulation, neovascularization, and remodeling are important components of graft incorporation.^30^ Whether these biological principles translate into improved durability following pectoralis major transfer remains unknown and requires further investigation.^30^

### Limitations

This report is limited to a technical description and surgical rationale. Clinical outcome data and long-term follow-up are not yet available. As such, definitive conclusions regarding durability, complication rates, or functional superiority over existing techniques cannot be made. Future studies evaluating clinical outcomes, complication profiles, and patient-reported measures will be necessary to validate the proposed advantages of this modification. In addition to absence of clinical outcome and biomechanical data, the technique is technically demanding and requires familiarity with scapulothoracic anatomy and anterior access to the inferior angle of the scapula. Risks specific to the fixation method include tunnel malposition, inadequate cortical purchase, implant-related complications, and potential scapular fracture. Furthermore, the procedure requires access to allograft tissue and suspensory fixation implants, which may limit generalizability across institutions. Potential allograft-related risks, including infection and immune reaction, should be discussed during consent.

## Conclusion

The single-incision modified pectoralis major transfer described in this report is designed to address several commonly reported limitations of traditional pectoralis major transfer techniques, including posterior exposure, exposed graft length, and intraoperative tensioning. Further clinical and biomechanical studies are required to evaluate functional outcomes, durability, complication rates, and construct performance.

## Disclaimers:

Funding: No funding was disclosed by the authors.

Conflicts of interest: The authors, their immediate family, and any research foundation with which they are affiliated have not received any financial payments or other benefits from any commercial entity related to the subject of this article.
