# Supplementary material for: Modified pectoralis major transfer: a single-incision technique for scapular winging
Source: JSES Rev Rep Tech. 2026 Aug 5;6(4):100845. doi: 10.1016/j.xrrt.2026.100845 (PMC13571266; doi:10.1016/j.xrrt.2026.100845)
Supplement: Supplementary file 1 [file mmc4.docx]

**Video Legend**

**Video 1.** Single-incision modified pectoralis major transfer with allograft augmentation and suspensory fixation.

The video demonstrates anterior-only exposure, selective sternal head release, integration of a tibialis anterior allograft within the transferred tendon, transosseous suspensory fixation at the inferior scapular angle, and controlled intraoperative tensioning.

**Video 2** Assessment of pectoralis major sternal head excursion following release.

After selective subperiosteal release of the sternal head at the humeral footprint, horizontal excursion of the tendon is assessed to confirm adequate mobilization without tension on the preserved clavicular head.

**Video 3** Incremental tensioning of the pectoralis major transfer using suspensory fixation.

The adjustable-loop suspensory device allows controlled, stepwise tensioning of the augmented pectoralis major tendon under direct visualization to reduce medial scapular winging while maintaining passive scapulothoracic mobility.
